# Is police investigation of rape biased by characteristics of victims?

**DOI:** 10.1016/j.fsisyn.2020.02.003

**Published:** 2020-02-22

**Authors:** Bjarte Frode Vik, Kirsten Rasmussen, Berit Schei, Cecilie Therese Hagemann

**Affiliations:** a Norwegian University of Science and Technology (NTNU), Department of Clinical and Molecular Medicine, P.B. 8905, N-7491, Trondheim, Norway; b Department of Research and Development, Psychiatry, St. Olavs Hospital, Trondheim University Hospital, Trondheim, Norway; c St. Olavs Hospital, Department of Neuropsychiatry, P.B. 3250 Torgarden, N-7006, Trondheim, Norway; d Norwegian University of Science and Technology (NTNU), Department of Psychology, 7491, Trondheim, Norway; e Norwegian University of Science and Technology (NTNU), Department of Mental Health, Faculty of Medicine and Health Sciences, Trondheim, Norway; f St. Olavs Hospital, Forensic Research Unit, Brøset, P.B. 1803 Lade, 7440, Trondheim, Norway; g Department of Public Health and Nursing, Norwegian University of Science and Technology, P.B. 8905, N-7491, Trondheim, Norway; h Department of Obstetrics and Gynecology St. Olavs Hospital, P.B. 3250 Sluppen, N-7006, Trondheim, Norway

**Keywords:** Rape, Police investigation, Victim vulnerability, Rape myths

## Abstract

**Aim:**

To explore differences in police investigations between cases of rape against women with and without vulnerability factors.

**Methods:**

Retrospective, descriptive study of cases of rape against women ≥16 years of age. Cases involving victims with and without vulnerability factors were compared regarding the quality of police investigation.

**Results:**

Vulnerability was present among 68% of the victims. Cases with vulnerable victims had an adjusted odds ratio for a low-quality police investigation of 2.1 (95% CI [1.0–4.4]) compared to cases where victims were non-vulnerable.

**Conclusions:**

Our results do not prove that rape myths existed among police officers. Our findings show a trend indicating that vulnerable victims may have been less prioritized compared to non-vulnerable victims. More studies are needed regarding how the police respond to rape complaints and to what degree police investigations are influenced by different characteristics of victims.

## 1 Introduction

A marked increase in police reported rapes has occurred in Norway over the past 25 years, from 400 cases per year in the 1990s to more than 1700 cases in 2017 [1]. The proportion of reported cases taken to trial and ending with conviction has decreased both nationally and internationally [[2], [3], [4], [5], [6]]. Several factors may be operating when attempting to understand this attrition of cases. Types of reported rapes have changed, and hence cases which were previously not acknowledged as rape are now being reported and subjected to police investigations [1,4,7]. This poses increased and updated demands regarding how the police respond in cases of rape [3,8,9]. As many studies have shown that the majority of cases are closed in the initial phase of the legal process [3,5,6,8,10] the research community has begun recognizing and studying police officers’ active involvement in the decision-making process [8]. Hence, how the police perceive a reported rape at the initial interview of the victim, is likely to inform the extent and type of supplementary investigation collected. Insufficiencies of steps taken initially may lead to evidence being overlooked, and cases wrongly dismissed [11]. The National Criminal Investigation Service in Norway (Kripos) reported in 2015 that 40% of police investigations of rape had been of poor quality and effectiveness [12]. Indications of low quality included insufficiencies in crime scene investigations and lack of securing a DNA profile from the alleged perpetrator. Research has confirmed that the forensic evidence collection is an important factor in the decision-making process of the prosecution of perpetrators [13]. An indication of suboptimal police investigation was shown in a previously published finding from the sexual assault center (SAC) at St. Olavs Hospital, Trondheim, Norway: Even though 55% of women reporting rape to the police had been medically examined at the SAC, including the collection of evidence kits, we found that the police submitted only 29% of the kits collected to further forensic analyses [14]. Other studies have disclosed similar results, but there is limited literature explaining why so many kits are never submitted to a crime laboratory in rape cases [[15], [16], [17], [18]]. Some explanations have been related to resource constraints, like staffing cuts in the police or insufficient capacity in crime labs [15].

What informs police decisions as to which investigational steps is poorly understood [8]. The decision-making process will depend on varying legislation and organization of the criminal justice systems of different countries [3]. Also within each country there are differences in the approach to sexual crimes between different police districts [4,15,19]. According to a study from the US much of the decision-making in the police is hidden from public scrutiny due to large amounts of discretion in the everyday routines of police officers. The destiny of a reported rape case is therefore often coincidental and dependent on how the police officer who takes notes chooses to interpret and document the reported incident [8]. Cases in which the police struggle at finding enough evidence may be dismissed without further investigation. As an example, a study from England and Wales described that police officers did not advance cases for prosecution when they believed that there were no realistic chances of securing convictions [20].

What influences the perceived process, may be based on stereotypes about what constitutes a “real rape” [7,21]. Such stereotypes may be termed “rape myths” and are used to describe factors which can bias the investigation of rape. Such myths may in particular be triggered by the characteristics of victims [22]. One study assessing official police records in rape cases found police notes which suggested that reported incidents were not “a real rape” because the victim was either a regular drug user, a sex worker, had reported rape before, was “mental”, or promiscuous [23]. The report concluded, in line with other studies, that the existence of such myths in the police environment may systematically predict case progression negatively [3,5,7,23,24]. An Australian criminological report from 2017 described several types of common misconceptions related to characteristics of the victim [25]: “People with disabilities are rarely victims of rape, and if subjected to rape they are not capable of relaying details about the incident”; “People with mental health problems often fabricate reports of rape”; “Intoxicated victims consent to sex but regret it afterwards and allege rape”. In a study from the UK, victims with mental health problems or learning disabilities, were found to be less likely to have their rape progressed through the criminal justice system [4]. Also, they found that victims who had previously reported one or more episodes of rape to the police tended to have their cases dismissed [4]. Several of the studies mentioned above claim that certain characteristics of rape victims may induce bias according to the “real rape” stereotype and hence lead to suboptimal police investigations.

In a previous study from the Trondheim sexual assault center (SAC) we found that a large proportion of victims did have characteristics which could potentially bias the investigations [26]. The observed characteristics included victims with intellectual and/or physical disabilities; as well as those reporting to have a history of mental health problems; substance abuse; and former sexual assault. The combined groups were classified as having one or more vulnerability factors. The aim of this study was to describe police investigations and assess differences between cases of rape in which the victims were characterized as being vulnerable compared with victims who did not have such characteristics.

## 2 Material and methods

### 2.1 Design, settings and sample

**Fig. 1:**
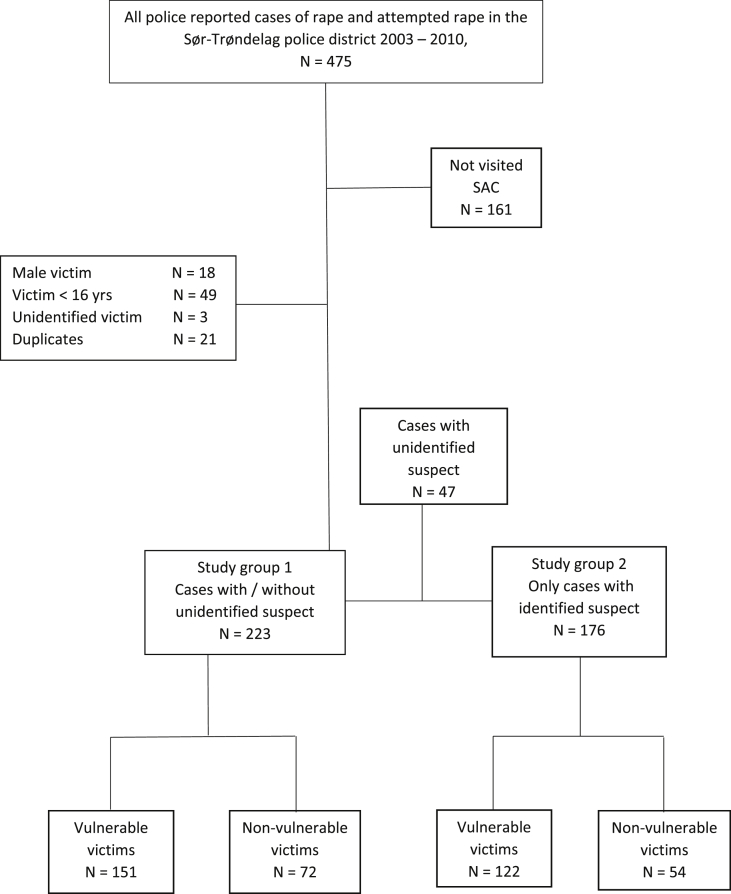
Flow chart for all included and excluded police recorded cases of rape and attempted rape in the Sør-Trøndelag police district during the period 2003–2010, and with corresponding records from the Trondheim SAC. Study group 1 includes all cases with/without unidentified suspect. Study group 2 includes only cases with identified suspect. The chart shows numbers of vulnerable and non-vulnerable victims in the two study groups.

This is a retrospective, descriptive study, based on merged data from police files and medical records in rape cases. During the observation period of this study (2003–2010) the Sør-Trøndelag Police District (STPD) in Norway covered a population of approximately 280 000 in the county of Sør-Trøndelag.^1^ Trondheim, the largest city in the region, was included, with 160 000 inhabitants at the time [27]. The only medical sexual assault center (SAC) in the district is located at St. Olavs hospital in Trondheim, and the service of this SAC, which provided the medical data for the study, is described in detail elsewhere [28]. All cases of rape and attempted rape against women ≥16 years of age reported to the STPD from July 1, 2003 to December 31, 2010 were registered. Cases were identified based on the former Norwegian penal code, §192, applicable until September 2015. According to this law a rape was defined as in the following abbreviated version: penetration of penis/finger/foreign object in vagina/anus, penis in mouth, masturbation, and coercion by means of violence, threats, or during impaired consciousness [29]. The following specific crime denominations were included, as described by the same former penal code, §192: Sections 1, 2 (rape), section 3 (aggravated rape) and section 4 (grossly negligent rape). Most of the cases included in our study were reported under sections 1, 2 (rape). Cases of attempted rape were also included but covered by another paragraph in the same penal code (§49). A total of 475 cases were reported to police during the period. Cases were excluded according to Fig. 1. Patients who did not visit the SAC (*n* = 161), male victims (*n* = 18), victims < 16 years of age (*n* = 49), unidentified victims (*n* = 3) and duplicate registrations (*n* = 21) were excluded, leaving 223 cases eligible for study group 1 (Fig. 1). For analyses which were relevant only in cases with identified suspect, we excluded all cases with unidentified suspect (n = 47), leaving 176 cases eligible for the comparisons (study group 2) (Fig. 1).

#### 2.1.1 Variables from police records and police investigational score (IS)

Data were collected from the police files regarding characteristics of suspects and victims, the reported incident, time from rape to police report, police investigations and legal outcome. In cases with more than one suspect information regarding the assumed most important suspect was recorded. Biological material from crime scene and suspects was collected by the police and found in police files. Information was collected about whether suspects and victims had formerly been registered as suspects/victims of crimes, and regarding time from police-reporting to legal decision-making. Police files provided data on to what extent different investigational procedures had been performed. We selected ten investigation variables for a police “investigational score” (IS), each of the ten variables counting a value of one score point (Table 2). We defined “high-quality investigation” for those with IS ≥ 7 of the 10 investigation variables. According to the Norwegian system, the police decide, in each case, whether a forensic medical record from the SAC is to be requested for further investigations, and we recorded whether the police had requested such medical forensic report from the SAC as one of our investigational factors. We also recorded whether the victim had contacted police before or after the SAC.

#### 2.1.2 Variables from SAC records

Documentation on medical examinations, forensic and toxicological analyses were retrieved from SAC records. Victim alcohol intake in relation to the assault was classified by three categories; “no intake”, “intake of <5 units of alcohol” and “intake of ≥5 units of alcohol/heavily intoxicated”. Time from assault to medical exam at the SAC was dichotomized to “< 24 h” or “≥ 24 h”, and results of toxicological samples from victims were noted as “no toxicological agents” or “≥ 1 toxicological agent”. Information was collected on whether the victim was suspicious of having been deliberately drugged prior to the assault. A victim was considered vulnerable if at least one of the following features was present: Intellectual and/or physical disabilities; history of present/former mental health problems; history of present/former alcohol/substance abuse; and former sexual assault. Information on whether victims had vulnerability or not was retrieved mainly from SAC records. For details, see Refs. [14,26,30,31].

#### 2.1.3 Data collection and merging

Data from police- and medical records were registered through two different web-based data collection systems developed and administered by the Unit of Applied Clinical Research at the Norwegian University of Science and Technology. In cases where SAC information was available and information in the police files was missing, victims’ medical records were the source of information. In case of discrepancy between the two data sources, information from the police files was used. Thereafter data were merged into one complete dataset.

### 2.2 Study approval

The study was approved by the Regional Committee for Medical and Health Research Ethics, the Norwegian Director General of Public Prosecutions and the Advisory Board on Secrecy and Research. The merging of data was also approved by the Norwegian Data Inspectorate.

### 2.3 Statistical analyses

For study group 1 (n = 223), we explored differences in victim- and assault characteristics (Table 1), and time between police report and legal decision between vulnerable and non-vulnerable victims, by using Pearson’s chi-square and Fisher’s Exact Tests (FET) as appropriate for the categorical variables. For the continuous variables we used Student’s T-test and Mann Whitney *U* test. Since four of our ten investigation variables were relevant only in cases where suspects were identified (n = 176, Study group 2), the 47 cases with unidentified suspect were excluded when exploring differences in police investigation. We used similar statistical methodology for Study group 2 as for Study group 1 when comparing vulnerable and non-vulnerable victims with regard to each of the ten police investigation variables (Table 2), for the IS, and for investigation quality (dichotomously separated into high- and low-quality with an IS ≥ 7) (Table 3). For comparing high and low-quality of police investigation, we used logistic regression analyses, calculating crude (cOR) and adjusted odds ratios (aORs) with corresponding 95% confidence intervals (CIs). We also examined the scores and quality of police investigations in cases with each of the vulnerability factors separately, and for those with more than one vulnerability factor. We had three statistical models: 1) adjusting for victim age (three-categorical) only; 2) adjusting for victim age, victim alcohol intake (three-categorical) and whether suspect was registered as a former suspect or not in the police files; and 3) adjusting for victim age, victim/suspect relationship (four categories), and reported penetration or not (by penis or object). Statistical significance was assumed when p < 0.05. Missing data were calculated but excluded when statistical tests were performed. Data analyses were performed using IBM SPSS Statistics for Windows, version 25.0.

**Table 1:** Victim – and assault characteristics among 223 victims of sexual assault and by vulnerability. Merged data from the Trondheim SAC and Sør-Trøndelag Police District.

Variable	Total N = 223 n (%)	Yes N = 151 n (%)	No N = 72 n (%)	*p*-value
**Victim age**				
Mean, median, SD, range	24.1, 20.9, 16-51	24.9, 21.3, ±8.5, 16-51	22.2, 19.9, ±7.2, 16-46	0.02^a^, 0.004^b^
**Victim origin, n=223**				
Western	210 (94)	141 (93)	69 (96)	
Non-Western	11 (5)	9 (6)	2 (3)	0.5^c^
Missing	2 (1)	1 (1)	1 (1)	
**Victim's occupation, n=223**				
Employed/student	159 (71)	95 (63)	64 (89)	
Unemployed	56 (25)	50 (33)	6 (8)	< 0.001^d^
Missing	8 (4)	6 (4)	2 (3)	
**Victim-suspect relationship, n=223**				
Partner	16 (7)	13 (9)	3 (4)	
Friend/family	82 (37)	58 (38)	24 (33)	
Casual contact	87 (39)	55 (36)	32 (44)	
Stranger	36 (16)	24 (16)	12 (17)	0.5^e^
Missing	2 (1)	1 (1)	1 (1)	
**Victim a former victim of a crime, n=217**				
No	79 (36)	41 (28)	38 (54)	
Yes	138 (64)	106 (72)	32 (46)	< 0.001^d^
**Victim a former suspect of a crime, n=217**				
No	131 (60)	76 (52)	55 (79)	
Yes	86 (40)	71 (48)	15 (21)	< 0.001^d^
**Victim alcohol intake, n=216**				
No intake	35 (16)	30 (21)	5 (7)	
<5 units	48 (22)	35 (24)	13 (18)	
≥5 units	133 (62)	80 (55)	53 (75)	0.009^f^
**Victim suspicious of being drugged, n=214**				
No	190 (89)	128 (87)	62 (94)	
Yes	24 (11)	20 (14)	4 (6)	0.15^d^
**Physical violence, n=223**				
None/verbal	28 (13)	20 (13)	8 (11)	
Light/moderate	139 (62)	93 (62)	46 (64)	
Severe	22 (10)	16 (11)	6 (7)	0.8^f^
Missing	34 (15)	22 (15)	12 (17)	
**Penetration, n=223**				
No penetration	30 (14)	18 (12)	12 (17)	
Penetration by penis or foreign object	164 (74)	114 (76)	50 (69)	0.39^d^
Missing	29 (13)	19 (13)	10 (14)	
**Place of assault, n=223**				
Private	149 (67)	106 (70)	43 (60)	
Public	73 (33)	44 (29)	29 (40)	0.1^d^
Missing	1 (0)	1 (1)	0 (0)	
**Time from assault to medical exam at the SAC, n=222**				
<24 h	162 (73)	114 (76)	48 (67)	
≥24 h	60 (27)	36 (24)	24 (33)	0.15^d^
**Tox. results victim, n=115**				
No tox. agents	34 (30)	16 (21)	18 (47)	
≥1 tox. agent	81 (70)	61 (79)	20 (53)	0.005^d^

a *t*-test.

b Mann Whitney *U* test (test for differences in median).

c Fischer’s Exact Test, df = 1.

d Chi square, df = 1.

e Fischer’s Exact Test, df = 3.

f Chi square, df = 2.

**Table 2:** Ten variables for police investigation in 176 police reported cases of rape, by vulnerability (excluding 47 cases with unidentified suspect). Merged data from the Trondheim SAC and Sør-Trøndelag Police District.

Variable	Total N = 176 n (%)	Yes N = 122 n (%)	No N = 54 n (%)	*p*-value
**Suspect interrogated, n = 171**				
No	16 (9)	13 (11)	3 (6)	
Yes	155 (91)	106 (89)	49 (94)	0.4^a^
**Victim interrogated, n=176**				
No	5 (3)	4 (3)	1 (2)	
Yes	171 (97)	118 (97)	53 (98)	1.0^a^
**Interrogation of other witnesses than victim and suspect, n=174**				
No	42 (24)	33 (28)	9 (17)	
Yes	132 (76)	87 (73)	45 (83)	0.1^b^
**Suspect arrested, n=160**				
No	101 (63)	73 (66)	28 (56)	
Yes	59 (37)	37 (34)	22 (44)	0.2^b^
**Police investigation of crime scene, n=176**				
No	73 (42)	53 (43)	20 (37)	
Yes	103 (59)	69 (57)	34 (63)	0.5^b^
**Police requested forensic medical record from SAC, n=176**				
No/missing	93 (53)	62 (51)	31 (57)	
Yes	83 (47)	60 (49)	23 (43)	1.0^b^
**Analysis of swabs and/or clothes collected from victim, n=158**				
No/missing	93 (59)	64 (58)	29 (60)	
Yes	65 (41)	46 (42)	19 (40)	0.8^b^
**Collection of swabs and/or clothes from suspect, n=176**				
No/missing	99 (56)	73 (60)	26 (48)	
Yes	77 (44)	49 (40)	28 (52)	0.3^a^
**Collection of biological material from crime scene, n=176**				
No/missing	90 (79)	99 (81)	40 (74)	
Yes	37 (21)	23 (19)	14 (26)	2.0^b^
**Suspect DNA profile taken, n=161**				
No	74 (46)	55 (44)	19 (37)	
Yes	87 (54)	55 (50)	32 (63)	0.2^b^

^1^ T-test.

^2^ Mann Whitney *U* test (test for differences in median).

a Fischer’s Exact Test (FET), df = 1.

b Chi square, df = 1.

**Table 3:** Vulnerability by police investigation quality in 176 cases of rape with identified suspect, crude and adjusted odds ratios for “low-quality investigation” are shown. Merged data from the Trondheim SAC and Sør-Trøndelag Police District.

Variable	Total, N = 176 n (%)	Police investigation score < 7, “low-quality investigation” N = 107 (61%)	Police investigation score ≥ 7, “high-quality investigation” N = 69 (39%)	Crude OR	OR Adjusted for Victim Age^a^	OR Adjusted for victim age, victim alcohol intake and suspect a former suspect^b^	OR Adjusted for victim age, victim/suspect relationship and penetration^c^
**At least 1 vulnerability, N = 176**							
Yes	122 (69)	79 (74)	43 (62)	1.7 (0.9–3.3)	1.8 (0.9–3.5)	2.0 (1.0–4.0)	2.1 (1.0–4.4)
No	54 (31)	28 (26)	26 (38)	Reference	Reference	Reference	Reference
**Disability, N** = **173**							
Yes	20 (12)	12 (11)	8 (12)	1.0 (0.4–2.5)	1.0 (0.4–2.5)	1.0 (0.4–2.9)	0.9 (0.3–2.5)
No	153 (88)	93 (89)	60 (88)	Reference	Reference	Reference	Reference
**Mental health problems, N** = **163**							
Yes	93 (57)	59 (60)	34 (53)	1.3 (0.7–2.4)	1.4 (0.7–2.6)	1.2 (0.6–2.4)	1.8 (0.9–3.6)
No	70 (43)	40 (40)	30 (47)	Reference	Reference	Reference	Reference
**Alcohol/drug abuse, N** = **174**							
Yes	24 (14)	14 (13)	10 (15)	0.9 (0.4–2.1)	1.0 (0.4–2.5)	0.8 (0.3–2.2)	1.4 (0.5–4.0)
No	150 (86)	92 (87)	58 (85)	Reference	Reference	Reference	Reference
**Former sexual assault, N** = **162**							
Yes	83 (51)	54 (55)	29 (45)	1.5 (0.8–2.8)	1.5 (0.8–2.9)	1.3 (0.7–2.7)	1.5 (0.7–3.0)
No	79 (49)	44 (45)	35 (55)	Reference	Reference	Reference	Reference
**Number of vuln.factors, N** = **176**							
0	54 (31)	28 (26)	26 (38)	Reference	Reference	Reference	Reference
1	47 (27)	32 (30)	15 (22)	2.0 (0.9–4.5)	2.0 (0.9–4.6)	2.5 (1.0–5.9)	2.4 (0.9–6.0)
>1	75 (43)	47 (44)	28 (41)	1.6 (0.8–3.2)	1.6 (0.8–3.5)	1.6 (0.7–3.6)	1.9 (0.9–4.4)

a Adjusted for age, 3-categorial (16–17 yrs, 18–24 yrs, ≥ 25 yrs).

b Adjusted for age, 3-categorical (16–17 yrs, 18–24 yrs, ≥25 yrs), victim alcohol intake (3-categorical), and suspect a former suspect (dichotome).

c Adjusted for age, 3-categorical (16–17 yrs, 18–24 yrs, ≥25 yrs), victim/suspect relationship (4-categorical: partner, friend/family, casual < 24 h, stranger) and penetration (dichotome).

## 3 Results

### 3.1 Victim- and assault characteristics (Table 1)

Among the 223 victims, 151 (68%) had at least one of the four vulnerability factors present: (Fig. 1, study group 1): 22 (10%) had intellectual and/or physical disability; 117 (53%) had a mental health problem; 29 (13%) had present or former alcohol or drug abuse; and 98 (44%) reported one or more prior incidents of sexual assault. Reporting more than one vulnerability factor occurred among 87 victims (39%). Table 1 presents victim- and assault characteristics by vulnerability among the 223 victims (Fig. 1). The mean age of victims was 24.9 (SD 8.5) years among the vulnerable and 22.2 (SD 7.2) years among the non-vulnerable (p = 0.02). The unemployment rate was higher among vulnerable victims than among the non-vulnerable (33% versus 8%, X^2^ = 16.5, df = 1, p = < 0.001). The victim was more frequently registered as a former victim of a crime in the police files, when the victim was vulnerable compared with the non-vulnerable cases (72% vs. 46%, X^2^ = 14.3, df = 1, p < 0.001). Additionally, a vulnerable victim was more often registered as a former suspect of a crime than a non-vulnerable victim (48% vs. 21%, X^2^ = 14.3, df = 1, p < 0.001). Among the 223 cases, 59 (27%) attended the SAC before police reporting, while 15 (7%) police-reported before attending the SAC. In 149 cases (67%) SAC- and police-reporting were on the same day (no difference between the groups of vulnerable and non-vulnerable). For further descriptions of victim- and assault characteristics, see Table 1.

### 3.2 Investigational actions performed (Table 2) and police investigation score (IS)

Table 2 shows the police investigations performed among the 176 cases with only identified suspects by vulnerability (Study group 2). The suspects were interrogated by the police in 106 (89%) cases with vulnerable victims and in 49 (94%) cases where victims were non-vulnerable (p = 0.4, FET). All but five of the 176 victims were interrogated; here, four of those not interrogated were vulnerable. In cases where the victim was vulnerable the police interrogated other witnesses than victim and suspect borderline less often than in cases where the victim was non-vulnerable (73% vs. 83%, X^2^ = 2.4, df = 1, p = 0.1). The suspect was arrested in 34% of the cases where victims were vulnerable and in 44% of cases involving non-vulnerable victims (X^2^ = 1.6, df = 1, p = 0.2). Police investigation of crime scene was conducted in 59% of the cases, irrespective of victim vulnerability. The police requested a forensic medical record from the SAC in 47% of the cases, and there were no differences between the two groups of victims. Biological samples collected from victims at the SAC (swabs and/or clothes) were sent for further analyses at the Institute of Forensic Medicine in Oslo, Norway, in 41% of the cases, regardless of which group of victims involved. Collection of swabs and/or clothes from suspect was performed in 40% of cases with vulnerable victims and in 52% of cases with non-vulnerable victims (p = 0.3, FET). Biological material was collected from the crime scene less often in cases with vulnerable victims than in cases with non-vulnerable victims (19% vs. 26%, X^2^ = 3.4, df = 1, p = 0.2). A DNA profile of the suspect was secured less often in cases where victims were vulnerable than in cases with non-vulnerable victims (50% vs. 63%, X^2^ = 2.3, df = 1, p = 0.2, respectively). When computing the investigation score points (IS) for each case we found a mean and median IS of 5.3 (SD 2.3) and 5.0, resp. in cases with vulnerable victims vs. 5.9 (SD 2.4) and 6.0, resp. in cases with non-vulnerable victims, the difference was borderline significant (p = 0.13 and p = 0.16, resp.).

### 3.3 High- and low quality of police investigation (Table 3)

Table 3 describes the proportions of low-quality- and high-quality police investigations between cases with vulnerable and non-vulnerable victims. Among cases involving vulnerable victims we found that a low-quality police investigation had been performed in 65% vs. in 52% of the cases involving non-vulnerable victims (p = 0.1) (Table 3). The aOR for a low-quality police investigation was 2.1 in cases with vulnerable victims, compared with cases with non-vulnerable victims, when adjusted for victim age, victim/suspect relationship and penetration (aOR = 2.1, 95% CI [1.0–4.4]). Table 3 also illustrates the proportions of low- and high-quality police investigations regarding each vulnerability subgroup. When comparing cases with victims with mental health problems to cases with victims without such problems, the aOR for having a low-quality police investigation was 1.8 (95% CI [0.9–3.6]), when adjusted for victim age, victim/suspect relationship and penetration. For those with one vulnerability factor only and for those with more than one vulnerability factor the aOR for low quality investigation var 2.4 and 1.9 resp. compared to those with no vulnerability (aOR = 2.4, 95% CI [0.9–5.9]) and (aOR = 1.9, 95% CI [0.9–4.4]).

### 3.4 Investigational results

For all the 223 cases including those with unidentified suspect, *rape* was reported in 95% of the cases and *attempted rape* in 5%, and there were no differences between the groups of vulnerable and non-vulnerable victims with regards to that variable (p = 0.8, FET). The mean time from police reporting until legal decision-making was 9 months (278 days) in the vulnerability group and 8 months (246 days) in the non-vulnerability group (p = 0.29). Investigations led to prosecution in 10% of the cases, regardless of victim vulnerability (p = 0.8, FET) (study group 1). Among the 176 suspects who were identified, 75% admitted sexual contact, and this phenomenon was less common if the victim was vulnerable than if she was non-vulnerable (69% vs. 88%, X^2^ = 5.8, df = 1, p = 0.03). Only two suspects admitted rape and five admitted culpability (study group 2).

## 4 Discussion

Based on merged records from the STPD and the Trondheim SAC, we found that 68% had at least one of the four vulnerability factors present. We found a borderline significant tendency that the police less often interrogated other witnesses than victim and suspect, less often arrested the suspect, less often collected biological material from the crime scene and a suspect DNA profile in cases with vulnerable victims compared to with non-vulnerable victims. According to our definition of “high investigational quality” (score ≥ 7 of the 10 investigational actions performed) we found that as many as 65% of cases involving vulnerable victims got a low-quality police investigation, vs. 52% of the cases involving non-vulnerable victims. The adjusted odds ratio for having a police investigation of low quality was more than doubled in cases with vulnerable victims compared with cases involving non-vulnerable victims. To our knowledge no previous studies have conducted a comprehensive comparison of police investigation in rape cases based on such differences in victim vulnerability.

What is considered optimal investigation in each individual case is difficult to assess, as police investigations are tailored to each case. Still, it seems that an average IS of only 5.5 out of 10 investigation actions, regardless of victim vulnerability being present or not, indicates a potential for improvement. In line with our findings, a new report by Amnesty International, concludes that police investigations of rape cases in Norway are not optimal [2].

Despite the differences described in police investigations between cases with vulnerable and non-vulnerable victims, we found no differences in the prosecution rates between the two groups of victims we compared. Case closure was decided by the police in 90% of the cases irrespective of victim vulnerability. In line with this, there are many studies showing that the majority of cases are closed early in the legal process [3,5,6,10]. However, we did not explore whether cases with vulnerable victims were closed in the initial phases or later on in the investigational process.

Our analyses showed that the police submitted only 41% of the available forensic evidence kits for further analyses, regardless of victim vulnerability being present or not. This finding confirms and highlights the topic of untested sexual assault kits as still being present at our police district during the observation time of our study. In a new study from Michigan which questioned why sexual assault kits so often were not submitted for testing, one explanation was found to be the presence of victim-blaming beliefs and rape myths among police officers. A suggested remedy to the problem was training police on the utility of forensic evidence and best practices in sexual assault investigations [15]. Some researchers claim that the perpetrator seldom denies sexual contact when swabs have been collected from the victim [32,33]. Hence, untested kits result in loss of possible valuable medico-legal evidence. Even more disturbing, however, regardless of vulnerability, we found that the police requested a medico-legal report from the SAC in less than half of the police reported cases. Among victims who visit a SAC there can be body injuries which are routinely documented by trained SAC personnel. In addition, the emotional status of victims visiting the SAC and other information reported from the rape incident are often well documented in SAC records. According to a Danish study, in half of the rape cases, the forensic clinical examination and medical report was important for the prosecuting authorities in the decision-making process [13]. Hence, deciding to collect only 47% of the medical reports, could mean loss of important evidence.

We found that suspects admitted sexual contact in 75% of the cases, and that they did so significantly less often in the cases with vulnerable victims than in cases with non-vulnerable victims. By admitting sexual contact, the suspect implies that nothing criminal has occurred, only acts of consensual sex. The finding that less suspects admitted sexual contact in cases with vulnerable victims than in cases involving non-vulnerable victims could be interpreted as suspects’ strategies and anticipations of whom the police are most likely to believe. Suspects may expect denial of any sexual contact as a more credible and respected scenario when it comes to victims with vulnerabilities than victims without. Nevertheless, almost none of the suspects admitted rape or culpability, regardless of victim vulnerability or not.

Among all the 223 women who reported rape to the police we found that a high proportion of them already had been registered in the police files as either a former victim and/or a former suspect of a crime. It was significantly more often so for the vulnerable victims than for the non-vulnerable. This indicates that there may be a link between involvement in criminal activity, either as a victim, as a suspect or both, and being a rape victim. In line with our findings, a report from the police district in the Norwegian capital Oslo (OPD) concluded that a population of women who have reported rape to the police are far more victimized but also more victimizing than the general population [34]. The OPD report mentioned individual differences in types of former criminal activity involvement within the sample of victims reporting rape to the police, but did not describe differences in police investigation depending on pattern of vulnerability among victims [34]. We did not find any significant risk for low-quality police investigation among those with prior or current drug abuse, or those with prior sexual assault compared to those without these vulnerability factors. However, others have shown an association between re-reporting of rape and early dismissal from police investigation [4]. When we explored the investigation regarding cases with victims with mental health problems we found a borderline significant aOR of 1.8 for low-quality police investigation compared to cases with victims without recorded mental health problems. This finding could be interpreted as a consequence of possible rape myths among investigating police officers towards victims with this specific vulnerability.

The data of this study are almost a decade old and hence they do not necessarily reflect more updated versions of how the police investigate rape in cases where victims have increased vulnerability. One example of progress was when the Norwegian government in 2015 changed the legislation regarding interrogations in cases where children and other especially vulnerable victims (adults with intellectual disability) were subjected to serious crimes. The purpose was to secure that interrogations were facilitated and adapted to the needs of these groups of victims, and thereby to strengthen their legal rights [35]. Theoretically, similar adaptions could have been implemented regarding interrogations and handling of other vulnerable groups of rape victims as well, for example those with mental health problems and/or alcohol or substance abuse. In January 2019, The Director General of Public Prosecutions in Norway announced a new review of the quality of police investigations in rape cases in 2020, based on recent disclosures of sub-optimal police work [36]. In other words, there is still a potential for improvements and a political wish to improve.

Some argue that the influence of rape myths on rape investigations may not be as pronounced as previously assumed [37]. They explain this through an increased public awareness of rape myths and the implementation of multidisciplinary rape response teams throughout the US and in many European countries [38,39]. In accordance with this, a UK study has shown increased likelihood of case progression in police districts which adhered tightly to a so-called “victim focused approach”. This approach emphasizes on believing victims from when they report and supporting them to remain in the criminal justice system. The idea is also to actively involve multidisciplinary links along with the ongoing police investigation, such as Sexual Assault Referral Centers (SARCs), Independent Sexual Violence Advisors (ISVAs), specialist sexual violence services, and health sector [4].

Furthermore, it is natural to think of non-vulnerable victims as having a stronger ability than the vulnerable to stand up for their rights and advocate for themselves. Hence this may contribute to higher quality of investigations among the more resourceful victims. Another UK study describes a category of young vulnerable women involved in gang culture and how they are used to being exposed to threats and intimidations [40]. When these women choose to report a rape to the police, they may have challenges already in terms of reduced victim credibility. Some may not have the courage or communicational skills to give honest answers during police interrogations. The risk of withdrawing the complaint is also high in such cases.

This study has several limitations. The data were collected retrospectively which means that information has not been systematically collected in a research context using standardized case report forms. Our data regarding police investigations were limited as we were not given access to original logs or notes in the police files, only to official records. We only had access to files from one out of the 27 police districts in Norway, limiting the national generalizability of our findings and possible identification of victims being a former victim/suspect in another district. The reliability of the data in a study like this is dependent on the accuracy by which both police officers and medical staff have recorded in each case.

When designing this study, all victims who had not visited the SAC were excluded (n = 161). This was done mainly for two reasons: Firstly, a sufficient and proper registration of vulnerability in victims was found only in SAC records. Secondly, in order to evaluate the quality of police investigations, we needed information regarding to what extent the police had utilized medical information in the process. A previous study from the same SAC showed, however, that one third of the police-reported rapes had occurred in rural areas, whereas the remaining had taken place in or near the city of Trondheim [14]. Among the former, 42% had attended the SAC vs. 61% among the latter. This implies that our exclusion of those who had not visited the SAC reduced the representability of cases from rural communities, compared to cases from the urban area. The low SAC attendance of victims of rapes perpetrated outside the urban area, may partly be explained by geographical reasons; distance may feel disconcerting. The geographical differences in forensic examination raises the question of whether health services and consequently also police investigations in rape cases are less adequate in rural areas than in the city.

Many of our findings are not statistically significant, and some variables have rather small sample size, which can lead to problems in determining significant associations. Further, when analyzing our findings of less thorough police investigations in cases involving vulnerable victims than in cases involving non-vulnerable victims, we point to rape myths among police officers as a possible explanation. This could, by some readers, be evaluated as a simplified and speculative interpretation. The report by the OPD mentioned above refers to specific victim-related challenges which often make rape case investigations extremely difficult. Typically, victims often lack the will or courage to cooperate, either by withdrawing their complaints or choosing not to show up for interrogations. Such challenges are sometimes seen even in cases where the police have decided to prosecute. These examples do not necessarily support the theory that rape cases have a low priority or that case closures result from rape myths in a preconceived police environment [34]. In order to obtain more accurate knowledge of how police officers think regarding different groups of rape victims, more qualitative studies based on interviews should be conducted on this topic in the future.

Our study also has strengths: Having access to sensitive police data for research purposes is uncommon in Norway and makes our findings an important source of information about how the police process cases of sexual violence in a Norwegian sample. Few prior studies have described such in-depth comparisons of police investigations of sexual assault cases depending on levels of vulnerability in victims. It is a unique strength of the study that we have merged information from police records and the SAC, both by enriching the variety of data available, and as information about how results from the forensic medical exams are being used in police investigations of rape crimes. The large number of variables and a long observation period of more than seven years also strengthen the study.

## 5 Conclusions

Previous literature has suggested that myths and misconceptions about sexual offending among law enforcement personnel can predict case progression negatively. Our results do not prove that rape myths existed among police officers at the STPD during the observation period of this study. Our findings do, however, show a trend indicating that vulnerable victims may have been less prioritized compared to non-vulnerable victims with regards to police investigations of rape. More studies are needed in the future regarding how the police respond to rape complaints in general, and on to what degree police investigations are influenced by different characteristics of victims.

## Funding

This work was supported by the 10.13039/501100011769Department of Research and Development, Psychiatry, St. Olavs Hospital, Trondheim University Hospital, Trondheim Norway.

## CRediT authorship contribution statement

**Bjarte Frode Vik:** Writing - original draft. **Kirsten Rasmussen:** Writing - original draft. **Berit Schei:** Writing - original draft. **Cecilie Therese Hagemann:** Writing - original draft.

## Declaration of competing interest

None.

## Footnotes

1 In a police reform conducted by the Norwegian government in 2016 the number of police districts in Norway was reduced from 27 to 12. Sør-Trøndelag Police District was merged with Nord-Trøndelag Police District into Trøndelag Police District. The two counties Sør-Trøndelag and Nord-Trøndelag were formally merged in 2018 into Trøndelag county.
